# (*E*)-3-(3-Bromo­phen­yl)-1-(4-methyl­phenyl)prop-2-en-1-one

**DOI:** 10.1107/S1600536808034867

**Published:** 2008-10-31

**Authors:** Hongqi Li, B. K. Sarojini, C. G. D. Raj, L. N. Madhu, H. S. Yathirajan

**Affiliations:** a Key Laboratory of Science & Technology of Eco-Textiles, Ministry of Education, College of Chemistry, Chemical Engineering & Biotechnology, Donghua University, Shanghai 201620, People’s Republic of China; b Department of Chemistry, PA College of Engineering, Nadupadavu, Mangalore 574 153, India; c Department of Studies in Chemistry, University of Mysore, Manasagangotri, Mysore 570 006, India

## Abstract

The title compound, C_16_H_13_BrO, was synthesized from the reaction of 3-bromo­benzaldehyde and 4-methyl­acetophenone in the presence of KOH. The mol­ecule adopts an *E* configuration with respect to the C=C double bond of the propenone unit. The dihedral angle formed by the aromatic rings is 46.91 (14)°. The crystal structure is stabilized by Br⋯Br inter­actions [3.4549 (11) Å].

## Related literature

For the properties and applications of chalcones, see: Pandey *et al.* (2005 ▶); Conti (2006 ▶); Lawrence *et al.* (2001 ▶); Nielsen *et al.* (2005 ▶); Dominguez *et al.* (2005 ▶). For related structures, see: Sarojini *et al.* (2007 ▶) and references cited therein.
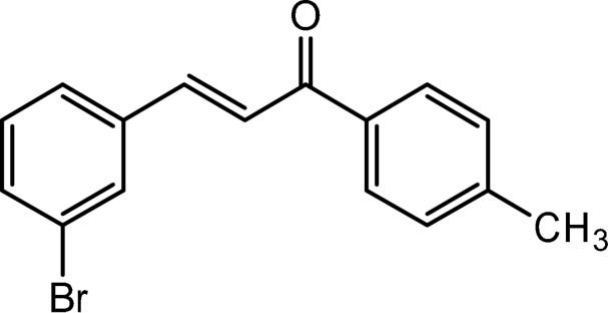


## Experimental

### 

#### Crystal data

C_16_H_13_BrO
*M* *_r_* = 301.17
Triclinic, 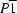

*a* = 5.8984 (16) Å
*b* = 7.3015 (19) Å
*c* = 15.559 (4) Å
α = 83.461 (5)°
β = 87.860 (4)°
γ = 88.446 (5)°
*V* = 665.1 (3) Å^3^
*Z* = 2
Mo *K*α radiation
μ = 3.08 mm^−1^
*T* = 273 (2) K
0.12 × 0.10 × 0.06 mm

#### Data collection

Bruker SMART CCD area-detector diffractometer
Absorption correction: multi-scan (*SADABS*; Sheldrick, 2004 ▶) *T*_min_ = 0.709, *T*_max_ = 0.837
3407 measured reflections
2312 independent reflections
1457 reflections with *I* > 2σ(*I*)
*R*_int_ = 0.049

#### Refinement

*R*[*F* ^2^ > 2σ(*F* ^2^)] = 0.058
*wR*(*F* ^2^) = 0.168
*S* = 1.00
2312 reflections
163 parameters
H-atom parameters constrained
Δρ_max_ = 0.57 e Å^−3^
Δρ_min_ = −0.44 e Å^−3^

### 

Data collection: *SMART* (Bruker, 2001 ▶); cell refinement: *SAINT* (Bruker, 2001 ▶); data reduction: *SAINT*; program(s) used to solve structure: *SHELXS97* (Sheldrick, 2008 ▶); program(s) used to refine structure: *SHELXL97* (Sheldrick, 2008 ▶); molecular graphics: *SHELXTL* (Sheldrick, 2008 ▶); software used to prepare material for publication: *SHELXTL*.

## Supplementary Material

Crystal structure: contains datablocks global, I. DOI: 10.1107/S1600536808034867/rz2257sup1.cif

Structure factors: contains datablocks I. DOI: 10.1107/S1600536808034867/rz2257Isup2.hkl

Additional supplementary materials:  crystallographic information; 3D view; checkCIF report

## Figures and Tables

**Fig. 1.**
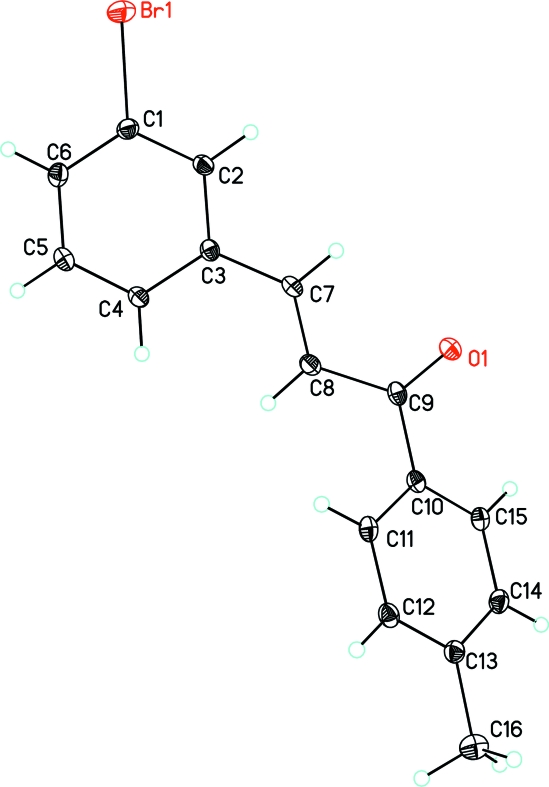
A view of the molecule of the title compound. Displacement ellipsoids are drawn at the 30% probability level and H atoms are shown as small spheres of arbitrary radii.

## supplementary crystallographic information

### Comment

Chalcones are a class of naturally occurring compounds with interesting biological properties such as cytotoxicity (Pandey *et al.*, 2005), antiherpes activity and antitumour activity (Conti, 2006) and may be useful for the chemotherapy of leishmaniasis among others (Lawrence *et al.*, 2001). Chalcone derivatives are also used as antibiotics (Nielsen *et al.*, 2005) and as anti malerials (Dominguez *et al.*, 2005). Recently, the crystal structures of some methyl- and bromo-substituted chalcones have been reported by our group (Sarojini *et al.*, 2007 and references cited therein). In a continuation of our studies, the title chalcone derivative was synthesized and its crystal structure is reported here.

The molecule of the title compound (Fig. 1) adopts an *E* configuration with respect to the C═C double bond of the propenone unit. The two aromatic rings are not coplanar, they dihedral angle they form being 46.91 (14) °. Molecular dimensions are unexceptional. The crystal structure is stabilized by Br···Br interactions occurring between centrosymmetrically-related molecules [Br1···Br1^i^ = 3.4549 (11) Å; symmetry code: (i) -x, 2-y, -z].

### Experimental

The title compound was prepared by adding 50% KOH (2.5 ml) to a solution of 4-methylacetophenone (1.34 g, 0.01 mol) and 3-bromobenzaldehyde (1.86 g, 0.01 mol) in ethanol (25 ml) at 273 K. The mixture was stirred for an hour and poured into crushed ice. The resulting yellow precipitate was collected by filtration and purified by recrystallization from ethanol. Single crystals suitable for X-ray analysis were grown by slow evaporation of an acetone solution (yield 80%). Analytical data: found (calculated): C %, 63.78 (63.81); H%, 4.30 (4.35).

### Refinement

All H atoms were placed at calculated positions and refined using the riding model approximation, with C—H = 0.93-0.96 Å and with *U*_iso_(H) = 1.2 *U*_eq_(C) or 1.5 *U*_eq_(C) for methyl H atoms.

### Crystal data

**Table:**

C_16_H_13_BrO	*Z* = 2
*M**_r_* = 301.17	*F*(000) = 304
Triclinic, *P*1	*D*_x_ = 1.504 Mg m^−^^3^
Hall symbol: -P 1	Melting point = 378–380 K
*a* = 5.8984 (16) Å	Mo *K*α radiation, λ = 0.71073 Å
*b* = 7.3015 (19) Å	Cell parameters from 1237 reflections
*c* = 15.559 (4) Å	θ = 2.6–23.7°
α = 83.461 (5)°	µ = 3.08 mm^−^^1^
β = 87.860 (4)°	*T* = 273 K
γ = 88.446 (5)°	Block, colourless
*V* = 665.1 (3) Å^3^	0.12 × 0.10 × 0.06 mm

### Data collection

**Table:**

Bruker SMART CCD area-detector diffractometer	2312 independent reflections
Radiation source: fine-focus sealed tube	1457 reflections with *I* > 2σ(*I*)
graphite	*R*_int_ = 0.049
φ and ω scans	θ_max_ = 25.1°, θ_min_ = 2.8°
Absorption correction: multi-scan (SADABS; Sheldrick, 2004)	*h* = −7→6
*T*_min_ = 0.709, *T*_max_ = 0.837	*k* = −8→6
3407 measured reflections	*l* = −18→18

### Refinement

**Table:**

Refinement on *F*^2^	Primary atom site location: structure-invariant direct methods
Least-squares matrix: full	Secondary atom site location: difference Fourier map
*R*[*F*^2^ > 2σ(*F*^2^)] = 0.058	Hydrogen site location: inferred from neighbouring sites
*wR*(*F*^2^) = 0.168	H-atom parameters constrained
*S* = 1.00	*w* = 1/[σ^2^(*F*_o_^2^) + (0.078*P*)^2^ + 0.164*P*] where *P* = (*F*_o_^2^ + 2*F*_c_^2^)/3
2312 reflections	(Δ/σ)_max_ < 0.001
163 parameters	Δρ_max_ = 0.57 e Å^−^^3^
0 restraints	Δρ_min_ = −0.44 e Å^−^^3^

### Special details

**Table:**

Geometry. All e.s.d.'s (except the e.s.d. in the dihedral angle between two l.s. planes) are estimated using the full covariance matrix. The cell e.s.d.'s are taken into account individually in the estimation of e.s.d.'s in distances, angles and torsion angles; correlations between e.s.d.'s in cell parameters are only used when they are defined by crystal symmetry. An approximate (isotropic) treatment of cell e.s.d.'s is used for estimating e.s.d.'s involving l.s. planes.
Refinement. Refinement of *F*^2^ against ALL reflections. The weighted *R*-factor *wR* and goodness of fit *S* are based on *F*^2^, conventional *R*-factors *R* are based on *F*, with *F* set to zero for negative *F*^2^. The threshold expression of *F*^2^ > σ(*F*^2^) is used only for calculating *R*-factors(gt) *etc*. and is not relevant to the choice of reflections for refinement. *R*-factors based on *F*^2^ are statistically about twice as large as those based on *F*, and *R*-factors based on ALL data will be even larger.

### Fractional atomic coordinates and isotropic or equivalent isotropic displacement parameters (Å^2^)

**Table:**

	*x*	*y*	*z*	*U*_iso_*/*U*_eq_	
Br1	0.20271 (11)	0.91736 (11)	0.07283 (4)	0.0905 (4)	
O1	−0.2214 (6)	0.7140 (5)	0.5453 (2)	0.0640 (10)	
C1	0.3158 (8)	0.9176 (7)	0.1855 (3)	0.0522 (13)	
C2	0.1824 (8)	0.8504 (7)	0.2552 (3)	0.0475 (12)	
H2	0.0413	0.8024	0.2469	0.057*	
C3	0.2584 (7)	0.8541 (6)	0.3379 (3)	0.0436 (11)	
C4	0.4711 (8)	0.9273 (6)	0.3481 (3)	0.0474 (12)	
H4	0.5246	0.9308	0.4033	0.057*	
C5	0.6015 (8)	0.9939 (7)	0.2770 (4)	0.0512 (13)	
H5	0.7434	1.0412	0.2844	0.061*	
C6	0.5234 (8)	0.9912 (7)	0.1949 (4)	0.0556 (13)	
H6	0.6100	1.0385	0.1466	0.067*	
C7	0.1059 (7)	0.7893 (6)	0.4108 (3)	0.0458 (12)	
H7	−0.0429	0.7673	0.3978	0.055*	
C8	0.1579 (8)	0.7590 (7)	0.4923 (3)	0.0519 (13)	
H8	0.3069	0.7715	0.5079	0.062*	
C9	−0.0197 (8)	0.7046 (6)	0.5605 (3)	0.0476 (12)	
C10	0.0571 (7)	0.6411 (6)	0.6491 (3)	0.0433 (11)	
C11	0.2715 (8)	0.5634 (7)	0.6637 (4)	0.0502 (12)	
H11	0.3718	0.5491	0.6172	0.060*	
C12	0.3359 (8)	0.5073 (7)	0.7474 (4)	0.0518 (13)	
H12	0.4773	0.4506	0.7567	0.062*	
C13	0.1921 (8)	0.5347 (7)	0.8172 (3)	0.0503 (12)	
C14	−0.0198 (8)	0.6113 (7)	0.8023 (3)	0.0544 (13)	
H14	−0.1188	0.6276	0.8489	0.065*	
C15	−0.0881 (8)	0.6646 (6)	0.7191 (3)	0.0468 (12)	
H15	−0.2322	0.7164	0.7101	0.056*	
C16	0.2693 (12)	0.4783 (10)	0.9080 (4)	0.0823 (19)	
H16A	0.1875	0.5502	0.9474	0.123*	
H16B	0.4289	0.4988	0.9104	0.123*	
H16C	0.2405	0.3499	0.9240	0.123*	

### Atomic displacement parameters (Å^2^)

**Table:**

	*U*^11^	*U*^22^	*U*^33^	*U*^12^	*U*^13^	*U*^23^
Br1	0.0839 (5)	0.1354 (8)	0.0529 (5)	−0.0024 (4)	−0.0134 (3)	−0.0102 (4)
O1	0.043 (2)	0.084 (3)	0.064 (2)	−0.0043 (17)	−0.0094 (17)	−0.002 (2)
C1	0.050 (3)	0.063 (3)	0.044 (3)	0.006 (2)	−0.003 (2)	−0.005 (2)
C2	0.038 (2)	0.053 (3)	0.052 (3)	0.001 (2)	−0.007 (2)	−0.009 (2)
C3	0.037 (2)	0.042 (3)	0.052 (3)	0.002 (2)	0.000 (2)	−0.009 (2)
C4	0.039 (3)	0.050 (3)	0.055 (3)	−0.001 (2)	−0.005 (2)	−0.015 (2)
C5	0.039 (3)	0.048 (3)	0.068 (4)	−0.002 (2)	−0.004 (3)	−0.012 (3)
C6	0.048 (3)	0.060 (3)	0.057 (3)	0.001 (2)	0.007 (2)	−0.004 (3)
C7	0.036 (2)	0.045 (3)	0.059 (3)	−0.002 (2)	−0.009 (2)	−0.012 (2)
C8	0.043 (3)	0.058 (3)	0.056 (3)	−0.007 (2)	−0.007 (2)	−0.010 (3)
C9	0.042 (3)	0.039 (3)	0.063 (3)	−0.007 (2)	0.001 (2)	−0.009 (2)
C10	0.037 (2)	0.036 (3)	0.057 (3)	−0.0047 (19)	−0.003 (2)	−0.005 (2)
C11	0.039 (3)	0.049 (3)	0.062 (3)	−0.003 (2)	0.011 (2)	−0.010 (3)
C12	0.039 (3)	0.046 (3)	0.068 (4)	0.000 (2)	−0.004 (3)	0.003 (3)
C13	0.049 (3)	0.047 (3)	0.053 (3)	−0.007 (2)	0.000 (2)	−0.001 (2)
C14	0.047 (3)	0.062 (3)	0.054 (3)	−0.003 (2)	0.011 (2)	−0.008 (3)
C15	0.038 (2)	0.043 (3)	0.058 (3)	−0.002 (2)	0.005 (2)	−0.005 (2)
C16	0.085 (4)	0.091 (5)	0.069 (4)	0.000 (4)	−0.011 (4)	0.002 (4)

### Geometric parameters (Å, °)

**Table:**

Br1—C1	1.899 (5)	C8—H8	0.9300
O1—C9	1.220 (6)	C9—C10	1.486 (7)
C1—C2	1.368 (7)	C10—C15	1.383 (7)
C1—C6	1.370 (7)	C10—C11	1.388 (6)
C2—C3	1.382 (6)	C11—C12	1.384 (7)
C2—H2	0.9300	C11—H11	0.9300
C3—C4	1.398 (6)	C12—C13	1.382 (7)
C3—C7	1.464 (7)	C12—H12	0.9300
C4—C5	1.374 (7)	C13—C14	1.373 (7)
C4—H4	0.9300	C13—C16	1.510 (8)
C5—C6	1.377 (7)	C14—C15	1.380 (7)
C5—H5	0.9300	C14—H14	0.9300
C6—H6	0.9300	C15—H15	0.9300
C7—C8	1.309 (7)	C16—H16A	0.9600
C7—H7	0.9300	C16—H16B	0.9600
C8—C9	1.491 (7)	C16—H16C	0.9600
			
C2—C1—C6	122.1 (5)	C10—C9—C8	117.6 (4)
C2—C1—Br1	118.6 (4)	C15—C10—C11	119.2 (5)
C6—C1—Br1	119.3 (4)	C15—C10—C9	118.8 (4)
C1—C2—C3	119.6 (4)	C11—C10—C9	122.0 (4)
C1—C2—H2	120.2	C12—C11—C10	119.9 (5)
C3—C2—H2	120.2	C12—C11—H11	120.0
C2—C3—C4	118.7 (5)	C10—C11—H11	120.0
C2—C3—C7	117.9 (4)	C13—C12—C11	120.7 (4)
C4—C3—C7	123.3 (4)	C13—C12—H12	119.7
C5—C4—C3	120.4 (5)	C11—C12—H12	119.7
C5—C4—H4	119.8	C14—C13—C12	119.1 (5)
C3—C4—H4	119.8	C14—C13—C16	121.2 (5)
C4—C5—C6	120.4 (4)	C12—C13—C16	119.7 (5)
C4—C5—H5	119.8	C13—C14—C15	120.9 (5)
C6—C5—H5	119.8	C13—C14—H14	119.6
C1—C6—C5	118.7 (5)	C15—C14—H14	119.6
C1—C6—H6	120.6	C14—C15—C10	120.2 (4)
C5—C6—H6	120.6	C14—C15—H15	119.9
C8—C7—C3	126.6 (4)	C10—C15—H15	119.9
C8—C7—H7	116.7	C13—C16—H16A	109.5
C3—C7—H7	116.7	C13—C16—H16B	109.5
C7—C8—C9	120.6 (5)	H16A—C16—H16B	109.5
C7—C8—H8	119.7	C13—C16—H16C	109.5
C9—C8—H8	119.7	H16A—C16—H16C	109.5
O1—C9—C10	120.6 (5)	H16B—C16—H16C	109.5
O1—C9—C8	121.8 (5)		
			
C6—C1—C2—C3	−0.7 (7)	O1—C9—C10—C15	−26.2 (7)
Br1—C1—C2—C3	−178.1 (3)	C8—C9—C10—C15	152.7 (4)
C1—C2—C3—C4	0.1 (7)	O1—C9—C10—C11	155.4 (5)
C1—C2—C3—C7	177.1 (4)	C8—C9—C10—C11	−25.7 (6)
C2—C3—C4—C5	0.0 (7)	C15—C10—C11—C12	1.3 (7)
C7—C3—C4—C5	−176.8 (4)	C9—C10—C11—C12	179.6 (4)
C3—C4—C5—C6	0.6 (7)	C10—C11—C12—C13	−2.7 (7)
C2—C1—C6—C5	1.3 (8)	C11—C12—C13—C14	2.8 (7)
Br1—C1—C6—C5	178.7 (4)	C11—C12—C13—C16	−177.9 (5)
C4—C5—C6—C1	−1.3 (7)	C12—C13—C14—C15	−1.5 (7)
C2—C3—C7—C8	170.1 (5)	C16—C13—C14—C15	179.2 (5)
C4—C3—C7—C8	−13.1 (7)	C13—C14—C15—C10	0.1 (7)
C3—C7—C8—C9	176.0 (4)	C11—C10—C15—C14	0.0 (7)
C7—C8—C9—O1	−11.5 (7)	C9—C10—C15—C14	−178.4 (4)
C7—C8—C9—C10	169.6 (4)		
